# Prognostic value of texture analysis from cardiac magnetic resonance imaging in patients with Takotsubo syndrome: a machine learning based proof-of-principle approach

**DOI:** 10.1038/s41598-020-76432-4

**Published:** 2020-11-25

**Authors:** Manoj Mannil, Ken Kato, Robert Manka, Jochen von Spiczak, Benjamin Peters, Victoria L. Cammann, Christoph Kaiser, Stefan Osswald, Thanh Ha Nguyen, John D. Horowitz, Hugo A. Katus, Frank Ruschitzka, Jelena R. Ghadri, Hatem Alkadhi, Christian Templin

**Affiliations:** 1 Institute of Diagnostic and Interventional Radiology, University Hospital Zurich, University of Zurich, Zurich, Switzerland; 2 Acute Cardiac Care, Andreas Grüntzig Heart Catheterization Laboratories, Department of Cardiology, University Heart Center, University Hospital Zurich, University of Zurich, Raemistrasse 100, 8091 Zurich, Switzerland; 3 Institute for Biomedical Engineering, University and ETH Zurich, Zurich, Switzerland; 4 Department of Cardiology, University Hospital Basel, Basel, Switzerland; 5 Department of Cardiology, Basil Hetzel Institute, Queen Elizabeth Hospital, University of Adelaide, Adelaide, Australia; 6 Department of Cardiology, Heidelberg University Hospital, Heidelberg, Germany

**Keywords:** Cardiology, Medical research

## Abstract

Cardiac magnetic resonance (CMR) imaging has become an important technique for non-invasive diagnosis of takotsubo syndrome (TTS). The long-term prognostic value of CMR imaging in TTS has not been fully elucidated yet. This study sought to evaluate the prognostic value of texture analysis (TA) based on CMR images in patients with TTS using machine learning. In this multicenter study (InterTAK Registry), we investigated CMR imaging data of 58 patients (56 women, mean age 68 ± 12 years) with TTS. CMR imaging was performed in the acute to subacute phase (median time after symptom onset 4 days) of TTS. TA of the left ventricle was performed using free-hand regions-of-interest in short axis late gadolinium-enhanced and on T2-weighted (T2w) images. A total of 608 TA features adding the parameters age, gender, and body mass index were included. Dimension reduction was performed removing TA features with poor intra-class correlation coefficients (ICC ≤ 0.6) and those being redundant (correlation matrix with Pearson correlation coefficient r > 0.8). Five common machine-learning classifiers (artificial neural network Multilayer Perceptron, decision tree J48, NaïveBayes, RandomForest, and Sequential Minimal Optimization) with tenfold cross-validation were applied to assess 5-year outcome including major adverse cardiac and cerebrovascular events (MACCE). Dimension reduction yielded 10 TA features carrying prognostic information, which were all based on T2w images. The NaïveBayes machine learning classifier showed overall best performance with a sensitivity of 82.9% (confidence interval (CI) 80–86.2), specificity of 83.7% (CI 75.7–92), and an area-under-the receiver operating characteristics curve of 0.88 (CI 0.83–0.92). This proof-of-principle study is the first to identify unique T2w-derived TA features that predict long-term outcome in patients with TTS. These features might serve as imaging prognostic biomarkers in TTS patients.

## Introduction

Takotsubo syndrome (TTS) or “broken heart” syndrome is an acute heart failure condition^1,2^. Although TTS has been classically recognized as a relatively benign condition, patients with TTS experience a wide range of outcomes from benign to life-threatening^3,4^. Recent studies demonstrated that TTS patients may have outcomes comparable to patients with acute coronary syndrome (ACS)^4,5^.

Cardiac magnetic resonance (CMR) imaging has gained an emerging role in non-invasive TTS diagnostics^6^, and it is regarded as the gold standard for differentiation of TTS from acute myocardial infarction^7^. Cine imaging allows for visualization of regional wall motion abnormalities that are characteristic for the disease^1^. T2-weighted (T2w) studies are able to detect myocardial oedema, which is a feature in the acute phase of TTS representing reversible myocardial injury and which matches the distribution of left ventricular (LV) dysfunction^6,8^. Myocardial oedema has been reported to be associated with repolarization abnormalities represented as electrocardiographic (ECG) T-wave inversion in patients with TTS^9^. Beyond T2w images, T1-weighted images 10–15 min after the administration of gadolinium are acquired for assessment of late gadolinium enhancement (LGE). Still, it remains controversial whether and to which extent LGE is present in patients with TTS^1,8^. However, if present, LGE in TTS has been described as being less intense than in patients with myocardial infarction (MI)^6,10^. It was discussed that LGE in the sub-acute phase of TTS may be associated with severity and prolonged recovery from the disease^11^.

Radiomics represents an emerging field of imaging research being characterized by conversion of imaging data into a highly dimensional mineable feature space generated by automatic data characterization algorithms^12^. In this context, texture analysis (TA) refers to an objective and quantitative set of metrics quantifying the texture of images^13^. These metrics can be used for diagnosing abnormalities in images that may not be seen by the human eye^14,15^. Because handling of such large amounts of data requires new, non-conventional statistical approaches, machine learning algorithms need to be applied. They facilitate mining of large amounts of data for identification of meaningful patterns and relationships between variables, potentially giving rise to novel imaging biomarkers^13^.

While CMR imaging in TTS is accurate in making the diagnosis through assessment of regional and global LV dysfunction^16^, to the best of our knowledge, no TA study so far has evaluated the prognostic value of CMR imaging in patients with TTS. Given that the risk of adverse events in TTS has been associated with the extent and severity of LV dysfunction^5,17^, we hypothesized that TA of the LV myocardium in patients with TTS might disclose abnormalities being predictive of future events. Thus, the aim of the present study was to evaluate the prognostic value of TA in CMR imaging in patients with TTS by using machine learning.

## Methods

### Patients and inclusion criteria

Patients with TTS were enrolled from the International Takotsubo (InterTAK) Registry, which is an observational, prospective and retrospective registry established in 2011 at the University Hospital Zurich, as previously described^5,18^. TTS was defined based on the InterTAK Diagnostic Criteria^2^. We included 106 patients from the InterTAK Registry who underwent CMR imaging. Twenty-four of these 106 patients (23%) were excluded because CMR imaging was not performed during the acute or subacute phase of TTS (≤ 14 days after symptom onset). Nineteen (18%) patients were excluded because CMR did not include all required sequences (T2w, T1Gd), and 5 (5%) patients were excluded because T2w and/or T1Gd images were of non-diagnostic image quality. When eligibility for study inclusion was unclear, cases were reviewed by core members at the University Hospital Zurich in order to reach a consensus.

Finally, a total of 58 patients with a diagnostic CMR imaging examination including both T2w and LGE images performed within 14 days after disease onset were included from four different centers (Zurich, Switzerland; Basel, Switzerland; Heidelberg, Germany; and Adelaide, Australia). Clinical data including demographics, vital signs, cardiovascular risk factors, comorbidities, laboratory values, and results from ECG were collected from hospital databases. Morphological patterns of LV wall motion abnormality in TTS were classified as typical (apical ballooning) or atypical type (midventricular, basal, or focal ballooning)^5,19,20^. Follow-up information was obtained through either telephone interviews, clinical visits, or medical records. The study protocol was reviewed by the respective local ethics committees (Ethikkommission Zürich) or investigational review boards at each collaboration site. All methods were carried out in accordance with relevant guidelines and regulations. All experimental protocols were approved by the Ethikkommission Zürich. At all sites where informed consent was required, formal written consent was obtained from each patient.

### Study outcome

Clinical outcome measure included the occurrence of major adverse cardiac and cerebrovascular events (MACCE). MACCE was defined as a composite of death from any cause, myocardial infarction, stroke or transient ischemic attack, or recurrence of TTS. All included patients were evaluated for the presence of MACCE over a 5-year follow-up period after the TTS index event. The outcome was noted in binary form and was used as outcome measure for machine learning classification.

### Cardiac MR imaging protocol

CMR imaging was performed on both 1.5 (Intera/Achieva, Philips Healthcare, Best, The Netherlands; Avanto, Siemens Healthineers, Forchheim, Germany) and 3.0 T (Skyra, Siemens Healthineers) scanners. The standard protocol included: (1) ECG-gated balanced steady-state free precession (SSFP) cine images in four standard geometries (i.e., short axis, 2-chamber, 3-chamber, and 4-chamber view); (2) T2w triple short-tau inversion recovery images in short axis geometry; (3) LGE imaging 10–15 min after intravenous administration of a bolus of gadolinium-based contrast agent; 0.2 mmol/kg.

### Image postprocessing

All images were anonymized and stored in digital imaging and communications in medicine (DICOM) file format for further processing. Gray level normalization was performed between the mean and three standard deviations (“± 3σ” method) helping to correct for small technical variations^21^. Certain TA features require identical spatial resolution and pixel size to be comparable^15^. Thus, all images were rescaled to a uniform in-plane resolution of 0.390625 × 0.390625 mm^2^ applying a custom MATLAB script (MathWorks, Natick, USA). This resolution was chosen equal to a standard native resolution with a field-of-view of 200 × 200 mm^2^ and matrix size of 512 × 512, which was chosen in consistency with former studies in this field^15,22^.

### Texture analysis

TA was performed using a freely available software package (MaZda, version 4.6, Institute of Electronics, Technical University of Lodz, Lodz, Poland)^23^.

**Figure 1:**
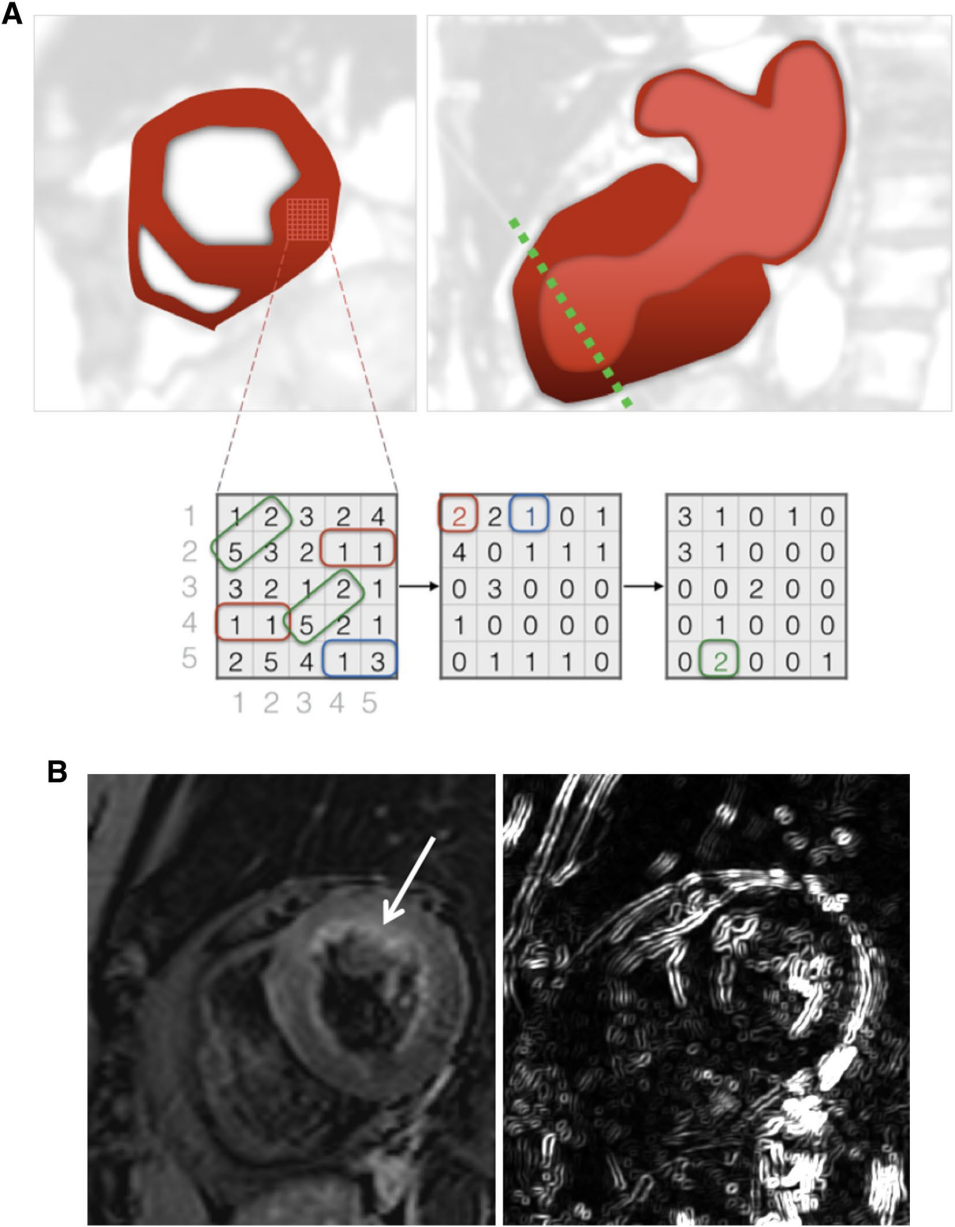
Cardiac Magnetic Resonance Imaging and Texture Analysis. (**A**) Schematic depiction of acute takotsubo syndrome with characteristic ballooning of the left ventricle in two-chamber longitudinal view *(top left image)*. The dotted line in green marks the plane for short axis cardiac magnetic resonance image acquisition *(top right)* at the point of maximal ventricular wall abnormality. After placement of a region-of-interest within the myocardium, a matrix of contained pixel intensities is generated *(bottom left)*. Derived from inter-pixel relationships, various texture analysis features can be computed. Grey-Level co-occurrence matrices (GLCM) for 0° *(bottom center)* and 45° *(bottom right)* are shown. Exemplarily, the combination of two consecutive pixels with value ‘1’ is found twice in the signal intensity matrix and therefore marked as ‘2’ in the GLCM (0°) matrix at position row = 1 and colums = 1 (red box). Similarly, two consecutive pixels with value ‘1’ and ‘3’ are found once and therefore marked as ‘1’ in the GLCM (0°) matrix at position row = 1 and column = 3 (blue box). The diagonal runs of values ‘5’ and ‘2’ are found twice in the signal intensity matrix and therefore marked as ‘2’ in the GLCM (45°) matrix at position row = 5 and column = 2 (green box). (**B**) 76-year old female patient with acute onset of takotsubo syndrome with basal oedema (*arrow*) in a fat saturated T2w short axis dark-blood image of the left ventricle. Image on the right depicts the corresponding map of the GLCM S(4,-4)DifVarnc feature.

Short-axis cine images showing the largest extent of LV dysfunction were identified for each patient. For TA, corresponding T2w and LGE images were used. On these images, polygonal regions-of-interest (ROIs) including the entire LV were drawn by MM and KK (*blinded for review*) for determining the inter-reader agreement of TA. One reader (MM) repeated ROI segmentation after two weeks for determining intra-reader agreement. An illustration of TA in TTS is provided in Fig. 1.

Overall, 608 (two times 304) TA features were computed per ROI originating from six main categories: (1) histogram (mean, variance, skewness, kurtosis), (2) grey-level co-occurrence matrix (GLCM) at five interpixel distances (angular second moment, contrast, correlation, entropy, sum entropy, sum of squares, sum average, sum variance, inverse different moment, difference entropy, difference variance), (3) run-length matrix (RLM) at four angles: horizontal, vertical, at 45° and at 135° (run-length non-uniformity, grey-level non-uniformity, long run emphasis, short run emphasis, fraction of image in runs), (4) absolute gradient (gradient mean, variance, skewness, kurtosis, non-zeros), (5) autoregressive model (teta 1 to 4, sigma), and (6) wavelet transform (Energy of wavelet coefficients in low-frequency sub-bands, horizontal high-frequency sub-bands, vertical high-frequency sub-bands, diagonal high-frequency sub-bands) (Table 1)^23^.

**Table 1:** Summary of all computed texture analysis categories with corresponding features.

Texture category	Texture feature
**Histogram**	Mean, variance, skewness, kurtosis
**Grey-level co-occurrence matrix (GLCM)** (computed for four directions [(a,0), (0,a), (a,a), (0,-a)] at five interpixel distances a = 1–5; 6 bits/pixel)	Angular second moment, contrast, correlation, entropy, sum entropy, sum of squares, sum average, sum variance, inverse different moment, difference entropy, difference variance
**Run-length matrix (RLM)** (computed for four angles [vertical, horizontal, 0°, and 135°]; 6 bits/pixel)	Run-length non-uniformity, grey-level non-uniformity, long run emphasis, short run emphasis, fraction of image in runs
**Absolute gradient** (4 bits/pixel)	Gradient mean, variance, skewness, kurtosis, and non-zeros
**Autoregressive model**	Teta 1–4, sigma
**Wavelet transform** (calculated for seven subsampling factors n = 1–7)	Energy of wavelet coefficients in low-frequency sub-bands, horizontal high-frequency sub-bands, vertical high-frequency sub-bands, diagonal high-frequency sub-bands

### Texture analysis feature selection

Feature selection was performed using the 608 TA features and adding the three non-TA features age, gender, and body mass index (BMI). All features showing a poor intra- and inter-reader agreement were removed from the present analysis. Intraclass correlation coefficients (ICC) were calculated for each pair of variables. According to Landis and Koch, ICCs of 0.61–0.8 were interpreted as substantial and 0.81–1.00 as excellent agreement^24^. TA features with ICC ≤ 0.6 were excluded from further analyses. Additionally, we used a correlation matrix discarding those TA features, which showed a high correlation defined by a Pearson correlation coefficient r > 0.8.

### Statistical analysis

Continuous variables were expressed as means ± standard deviations or medians with interquartile ranges (IQR), as appropriate. Categorical variables were provided as frequencies or percentages.

After feature selection, five commonly applied machine learning classifiers were used: Artificial Neural Network (ANN), decision tree classifier J48 (C4.5), NaïveBayes, RandomForest, and Sequential Minimal Optimization (SMO).

In detail, the ANN used is based on Multilayer Perceptron, a classifier that uses backpropagation to classify instances with n = *(number of attributes* + *classs)/2* hidden layers, a default learning rate of 0.3, and a momentum of 0.2. NaïveBayes represents a probabilistic classifier based on Bayes' theorem. RandomForest represents a meta-estimator that fits multiple decision tree classifiers on various sub-samples of the dataset and uses averaging to improve its predictive accuracy. J48 is based on C4.5 and generates a pruned decision tree. SMO implements sequential minimal optimization for training a support vector classifier.

In order to account for overfitting, we used tenfold cross validation splitting the dataset into ten parts randomly. During the course of ten iterations each part was once used for testing, while the remaining nine parts were used for algorithm training. Final results represented the averaged findings of all ten calculations. Machine learning classifier performance was compared by means of sensitivity, specificity, precision, recall, F-Measure, receiver operating characteristics (ROC) analysis with calculation of the area-under-the-curve (AUC), and precision recall curve (PRC).

Differences among single TA features between TTS patients with and without MACCE were compared using a Mann–Whitney U Test. Differences in AUC were compared according to Hanley and McNeil^25,26^. A two-tailed p value below 0.05 was considered to indicate statistical significance.

Data mining and machine learning algorithms were performed using open-source software (WEKA, University of Waikato, Waikato, New Zealand). All remaining statistical analyses were conducted using commercially available software (SPSS 23.0, IBM, Armonk, New York).

## Results

### Study population

Fifty-eight patients with the diagnosis of TTS and with CMR examination with diagnostic image quality were included in the present study. In this cohort, 56 (96.6%) were women and the mean age was 68.4 ± 11.8 years. Other baseline characteristics of these patients are summarized in Table 2. CMR imaging was performed within a median of 4 days (IQR, 2–6 days) after index TTS event. Five-year follow-up was obtained in all 58 patients. Long-term MACCE rate after 5-year follow-up was 10.3%.

**Table 2:** Baseline characteristics of patients with takotsubo syndrome.

Characteristics	N = 58
**Demographics**	
Female sex	56 (96.6)
Age, mean (SD), y	68.4 (11.8)
BMI, mean (SD), kg/m^2^	24.4 (4.3), n = 29
**Triggers**	
Physical trigger	20 (34.5)
Emotional trigger	24 (41.4)
Both emotional and physical trigger	3 (5.2)
No evident trigger	11 (19.0)
**Takotsubo type**	
Apical type	30 (51.7)
**Cardiac biomarkers on admission, median (IQR)**	
Troponin, fold ULN	8.0 (3.7–16.3), n = 53
Creatine kinase, fold ULN	1.20 (1.0–1.5), n = 40
**ECG on admission**	
ST-segment elevation	13 of 39 (33.3%)
**Vital signs, mean (SD)**	
Heart rate, beats/min	85.0 (19.7), n = 28
Systolic blood pressure, mmHg	131.1 (38.3), n = 24
Diastolic blood pressure, mmHg	80.2 (18.4), n = 21
**Hemodynamics, mean (SD)**	
Left ventricular ejection fraction, %	40.2 (10.2), n = 41
**Cardiovascular risk factors**	
Arterial hypertension	31 (53.4)
Current smoking	6 (10.3)
Diabetes mellitus	10 (17.2)
Hypercholesterolemia	21 (36.2)

Data are presented as number (percentage) of patients unless otherwise indicated.

*BMI* body mass index, *ECG* electrocardiogram, *IQR* interquartile range, *SD* standard deviation, *ULN* upper limit of the normal range.

### Dimension reduction

464 of the 611 (76%) texture features were removed because of low ICCs. Use of the correlation matrix to avoid redundancy led to a further dimension reduction to 10 TA features. These remaining features were exclusively derived from T2w images, while all T1Gd-derived features as well as all non-texture features were discarded due to poor reliability and/or redundancy. The ten selected TA features showed a substantial inter-reader (ICC 0.75 ± 0.1, range 0.7–0.99) and excellent intra-reader agreement (ICC 0.88 ± 0.1, range 0.7–0.99).

### 5-year MACCE classification results

After ten-fold cross validation, the machine learning classifier NaïveBayes showed a predictive ability of 5-year MACCE with a sensitivity of 82.9% (confidence interval (CI) 80–86.2), specificity of 83.7% (CI 75.7–92) and AUC of 0.88 (CI 0.83–0.92). While all four remaining classifiers showed higher sensitivities, both the specificity and the AUC were highest for the NaïveBayes classifier. Obtained precision was 0.88 (CI 0.83–0.92) and PRC area was 0.98 (CI 0.97–0.99). Detailed results are listed in Table 3.

**Table 3:** Detailed results of machine learning-based classification of 5-year major adverse cardiovascular and cerebral events [95% confidence interval].

Machine learning classifier	Sensitivity %	Specificity %	Precision	Recall	F-Measure	AUC from ROC curve analysis	PRC Area
ANN	85.2 [82.9–87.6]	17.4 [15–20]	0.94 [0.9–0.97]	0.85 [0.83–0.88]	0.9 [0.86–0.94]	0.79 [0.74–0.84]	0.94 [0.93–0.96]
J48 (C4.5)	85.1 [82.5–87.6]	17.3 [15–20]	0.94 [0.9–0.98]	0.85 [0.82–0.88]	0.9 [0.85–0.94]	0.51 [0.48–0.53]	0.84 [0.81–0.86]
NaïveBayes	82.9 [80–86.2]	83.7 [75.7–92]	0.88 [0.83–0.92]	0.83 [0.8–0.86]	0.89 [0.86–0.91]	0.88 [0.83–0.92]	0.98 [0.97–0.99]
RandomForest	89.4 [87.4–91]	31.7 [23.3–40]	0.98 [0.96–1]	0.89 [0.87–0.91]	0.96 [0.94–0.98]	0.8 [0.74–0.86]	0.94 [0.92–0.96]
SMO	90 [88.4–91.6]	16.7 [16.7–16.7]	1 [1–1]	0.9 [0.88–0.92]	1 [1–1]	0.5 [0.5–0.5]	0.83 [0.81–0.86]

*ANN* Artificial neural network (multilayer perceptron), *AUC* area-under-the-curve, *PRC* precision recall curve, *ROC* receiver operator characteristics, *SMO* sequential minimal optimization.

**Figure 2:**
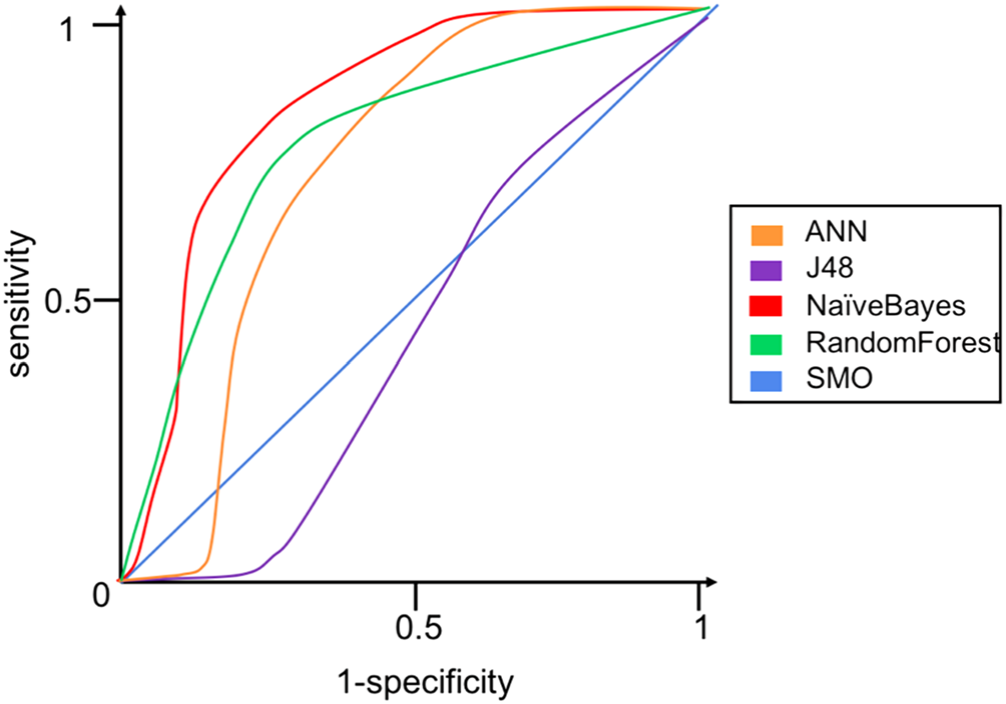
Receiver operator characteristics for texture analysis on T2-weighted cardiac magnetic resonance images with five different machine learning classifiers of 5-year major adverse cardiovascular and cerebral events in patients with takotsubo syndrome. Of note, the NaïveBayes classifier shows the highest area-under-the-curve (0.88).

Comparison of the AUC according to Hanley and McNeil^26^ showed significantly higher AUC values for the NaïveBayes classifier (0.88, 0.83–0.92) as compared to the RandomForest classifier showing the second highest AUC (0.8, 0.74–0.86])(*p* < 0.05). A graphical depiction of the ROC curves is provided in Fig. 2.

**Figure 3:**
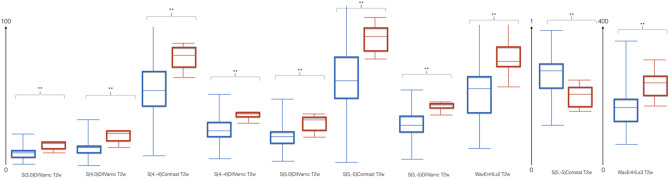
Box-Whisker plots of the ten selected texture analysis features. Dimension reduction yielded 10 TA features carrying prognostic information, which were all based on T2w images. Red: positive 5-year major adverse cardiovascular and cerebral events (MACCE), blue: negative 5-year MACCE. ** = *p* < .01.

The Mann–Whitney U test showed significant differences (*p* < 0.01) between the ten aforementioned TA features in TTS patients with positive and negative 5-year MACCE. Box-whisker plots of the selected TA features are shown in Fig. 3.

## Discussion

The present multicenter study identified for the first time TA features derived from T2w CMR images that predict long-term outcome in patients with TTS. A total of 304 texture features per CMR image and 3 non-texture features were analyzed, giving rise to a total of 35′438 ((2 × 304) + 3) × 58) values in the 58 TTS patients for classification of 5-year MACCE. We assessed the reproducibility as well as the redundancy of TA features by means of ICCs and a correlation matrix, respectively. After dimension reduction we identified 10 TA features carrying potential prognostic information in TTS.

While the exact underlying mechanism of TTS is unknown, it is suggested that TTS has a multifactorial etiology, involving the vascular, endocrine, and central nervous system^6^. Furthermore, it has been hypothesized that high intraventricular pressure may precipitate perfusion abnormalities and even regional myocardial ischemia^6^. Myocardial oedema has been shown to be a marker of abnormal systolic function and tissue damage in patients with TTS^6^. It can be detected by use of T2w CMR image sequences^27^ and the extent thereof is known to correlate with disease severity in terms of regional contractile disturbances as well as release of catecholamines and NT-proBNP in TTS^28^. In line with this, our study findings suggest a prognostic value of T2w-derived TA features in patients with TTS indicating a link between initial LV myocardial dysfunction and long-term outcome. These T2-related changes possibly reflect an inflammatory component and/ or increased vascular permeability^29,30^.

As mentioned above, the presence and extent of LGE in patients with TTS is still discussed controversially^1,6,8,10,11^, which is why we also included LGE images into our analyses. Interestingly, the dimension reduction approach discarded all TA features derived from LGE images—a finding contrary to studies discussing the presence and severity of LGE as a marker for poor prognosis.

As there is a known sex and age preference in TTS towards females between the ages of 62 and 76 years^31^ and evidence has accumulated that certain features including age, sex, and BMI are related to delayed TTS recovery^32^, we included also these three non-TA parameters to our analysis. However, our proof-of-principle analysis showed that these parameters did not carry prognostic information regarding the occurrence of 5-years MACCE. In our multicenter cohort, the predictive model was exclusively based on imaging. The ten TA features that enabled the machine learning based prediction of MACCE were all derived from higher level TA features of the grey-level co-occurrence matrix and the autoregressive model, whose characteristics are invisible to the human eye^15^.

### Study limitations

The following study limitations must be acknowledged. First, this was a retrospective analysis of prospectively acquired data from a multicenter trial. Due to the use of multiple scanners, image postprocessing with regard to pixel spacing was performed. Larger datasets are likely to improve the performance of supervised classifiers, decrease overfitting of the used algorithms and allow for implementation of deep learning, in which manual 2D image segmentation is no longer required. Second, the degree of myocardial edema detected by T2W images might change over time. Thus, our results need to be confirmed by future prospective studies. Finally, our study does not provide the causal link between abnormal LV texture and long-term outcome. Thus, prognostic implications based on our study results must be considered as hypothesis-generating. Prognostic implications of abnormal myocardial texture in TTS patients should be confirmed in future–ideally prospective–studies.

## Conclusions

**Figure 4:**
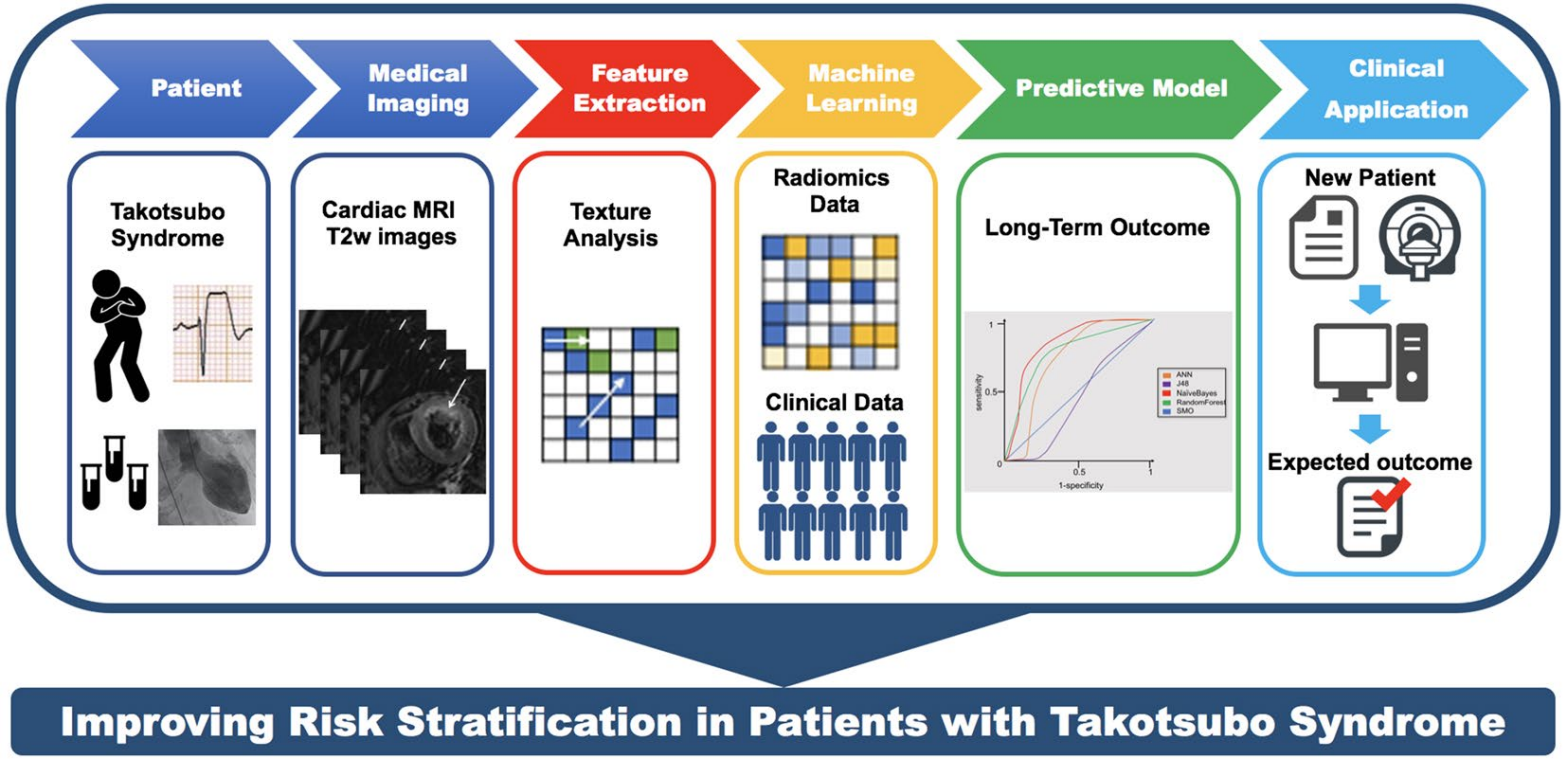
Improving risk stratification in patients with takotsubo syndrome. Cardiac magnetic resonance imaging-derived texture analysis features, identified through machine learning algorithms, have a prognostic value in patients with takotsubo syndrome. Thus, these features might serve as novel imaging biomarkers for risk stratification in takotsubo syndrome.

In the present proof-of-principle study, we demonstrated that selected TA features, exclusively derived from T2w CMR images and identified through machine learning, have potential prognostic long-term value in patients with TTS. Thus, they may serve as novel imaging biomarkers for risk stratification in patients with TTS (Fig. 4).

## Abbreviations

ACS: Acute coronary syndrome
ANN: Artificial neural network
AUC: Area-under-the-curve
BMI: Body mass index
CMR: Cardiac magnetic resonance
CI: Confidence interval
ECG: Electrocardiogram
ICC: Intraclass correlation coefficients
InterTAK: International Takotsubo
IQR: Interquartile ranges
GLCM: Grey-level co-occurrence matrix
LGE: Late gadolinium enhancement
LV: Left ventricle
MACCE: Major adverse cardiac and cerebrovascular events
MI: Myocardial infarction
PRC: Precision recall curve
RLM: Run-length matrix
ROC: Receiver operating characteristics
ROI: Regions-of-interest
SMO: Sequential minimal optimization
T2w: T2-weighted images
TA: Texture analysis
TTS: Takotsubo syndrome
